# Effect of Sieve Particle Size and Blend Proportion on the Quality Properties of Peeled and Unpeeled Orange Fleshed Sweet Potato Composite Flours

**DOI:** 10.3390/foods9060740

**Published:** 2020-06-04

**Authors:** Solomon Kofi Chikpah, Joseph Kudadam Korese, Oliver Hensel, Barbara Sturm

**Affiliations:** 1 Faculty of Organic Agricultural Sciences, Section of Agricultural and Biosystems Engineering, University of Kassel, Nordbahnhofstraße 1a., 37213 Witzenhausen, Germany; agrartechnik@uni-kassel.de (O.H.); barbara.sturm@uni-kassel.de (B.S.); 2 Faculty of Agriculture, Department of Food Science and Technology, University for Development Studies, Nyankpala Campus P.O. Box TL 1882, Ghana; 3 Faculty of Agriculture, Department of Agricultural Mechanisation and Irrigation Technology, University for Development Studies, Nyankpala Campus P.O. Box TL 1882, Ghana; jkorese@uds.edu.gh

**Keywords:** bioactive compounds, composite flour, functional properties, orange fleshed sweet potato, sieve particle size

## Abstract

Orange fleshed sweet potato (OFSP) has great potentials to improve the nutritional benefits of bakery products when processed into quality flour. This study investigated the effects of sieve particle sizes (250 μm and 500 μm) and flour blend proportions on the physicochemical, nutritional, functional and pasting properties of peeled and unpeeled OFSP composite flours. Peeled OFSP composite flours had significantly (*p* < 0.05) higher crude protein (CP), lightness (L*), oil absorption capacity (OAC) and water solubility (WS) but lower crude fiber (CF), bioactive compounds (except ascorbic acid), water absorption capacity (WAC) and swelling capacity (SC) than the unpeeled OFSP composite flours. The sieve particle size had no significant (*p* > 0.05) effect on nutritional and pasting properties. However, OFSP-based flours sieved with 500 μm mesh particle size had a significantly (*p* < 0.05) higher WAC and SC but a lower WS than corresponding 250 μm mesh flours. The proportions of flour blends greatly (*p* < 0.001) influenced all quality properties of OFSP composite flours. Generally, OFSP composite flours had higher CF, bioactive compounds, WAC, SC and WS, but lower CP, fat, OAC and pasting properties than wheat flour. The quality properties indicated that peeled and unpeeled OFSP flours sieved with a 250 μm or 500 μm mesh size have great potentials as ingredients in the bakery industry.

## 1. Introduction

The recent increasing trend of hunger and undernourishment in populations in the subregions of Africa, especially in Western Africa, has raised concern for timely intervention in order to achieve the set goal for zero hunger by 2030 [1]. Hunger is a major cause of micronutrient deficiencies such as vitamin A and iron deficiencies, which affect largely children and women of reproductive age, particularly in Sub Saharan Africa (SSA). According to the African Child Policy Forum (ACPF) [2], hunger does not only contribute to about 45% of childhood deaths in Africa, but also promotes poor health and poverty and reduces productivity and economic growth.

Sweet potatoes (*Ipomoea batatas* L. Lam) are an important source of nutrients and livelihood in developing countries like in SSA. They are reported as the third most important root crop after cassava and potato [3]. The orange fleshed sweet potato (OFSP) root is rich in health-promoting compounds such as carotenoids, anthocyanin, polyphenols and ascorbic acid [3,4], carbohydrates, dietary fiber and essential minerals [4,5]. The processing of OFSP roots into flour would not only extend its shelf life, reduce its bulkiness and diversify its application in food [6], but would also reduce wheat flour importation and create jobs for farmers and actors in the value chain as well as improve nutrition [4].

Several studies on OFSP flour production have largely focused on effects of different cultivars, pretreatments and drying methods [3,7]. The processing of OFSP for dehydration involves peeling which is very tedious, causes root loss and influences the chemical composition of the flour [8]. In addition, milling and sieving influence flour particle size which can further affect its functional properties [9]. The combination of different flours may also alter nutritional, physicochemical and functional properties of the composite flour as well as the quality characteristics of the final developed food product. According to Martins et al. [10], the analyses of flour blends provide necessary information for its practical application in the food industry for products development. Therefore, this study aimed to investigate the effects of sieve particle sizes and proportions of flour blends on the physicochemical, nutritional, functional and pasting properties of peeled and unpeeled OFSP composite flours.

## 2. Materials and Methods

### 2.1. Sweet Potato Samples

About 200 kg of non-infested, fresh and mature OFSP roots (*Ipomoea batatas* L.cv.CRI-Apomuden) harvested 100 days after planting were purchased from a commercial farm in Dambai in the Oti Region of Ghana. The roots were immediately transported to a laboratory, stored in a dry cool place and processed within five days after harvesting.

### 2.2. Orange Fleshed Sweet Potato Flour Processing

**Figure 1:**
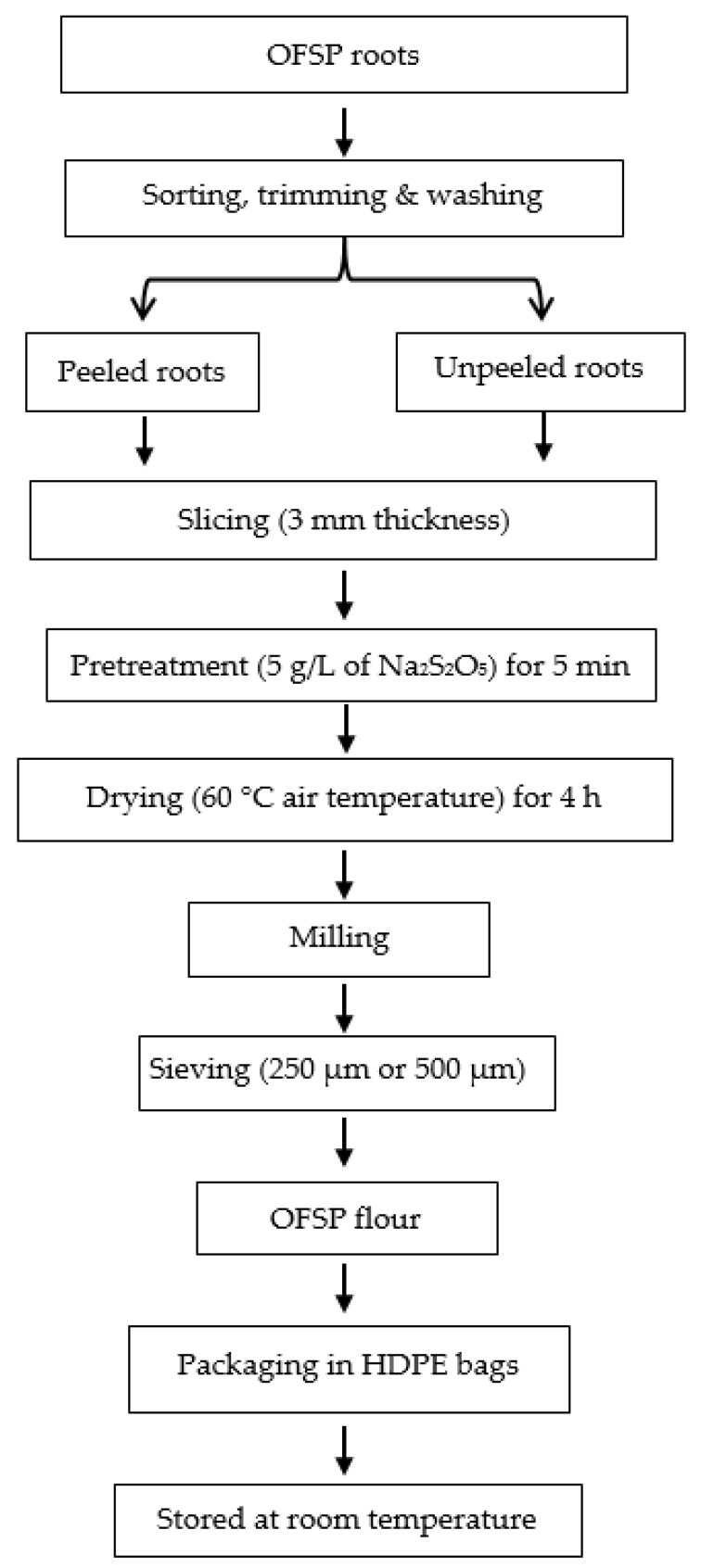
Flow chart for processing peeled and unpeeled orange fleshed sweet potato (OFSP) flours. HDPE represent high density polyethylene.

OFSP roots were processed into peeled and unpeeled flours as illustrated in Figure 1. Briefly, the roots were sorted, trimmed, washed manually with clean water and divided into two equal halves. One portion was peeled manually and sliced into uniform sizes of 3 mm thickness [4] using an electrical slicing machine (Ritter E16, Ritterwerk GmbH, Gröbenzell, Germany). The slices were soaked in 5 g/L sodium metabisulfite solution for 5 min and excess water was drained from the samples for 5 min as described by Hamed et al. [8] with slight modification. About 300 g of pretreated slices were spread out in a single layer on perforated trays and dried at 60 °C air temperature [11] using “Hohenheim HT mini” cabinet dryer (*Innotech-ingenieursgesellschaft* GmbH, Altdorf, Germany). The fresh slices had an initial moisture content of 3.34 g water per g dry matter (g_w_/g_DM_) and were dried to below 0.1 g_w_/g_DM_, packed into high-density polyethylene bags and stored in a dry cabinet at 25 °C ± 2 until all the drying was done.

The peeled OFSP dried slices were milled into flours, divided into two equal halves and sieved with either 250 µm or 500 µm mesh particle size (Model: Setaccio Di Prova, Laboratory test sieve, Milano, Italy). With the exception of peeling, the above procedure was used to produce unpeeled OFSP flour from the second portion of OFSP roots.

### 2.3. Preparation of Composite Flour of Different Particle Sizes

Each of the peeled and unpeeled OFSP flours (250 µm and 500 µm sieve particle sizes) were used to replace wheat flour at the rates of 0%, 10%, 20%, 30%, 40%, 50%, 60%, 70%, 80%, 90% and 100%. Each composite flour (500 g) prepared was packed in well-labeled high-density polyethylene bags and stored at 4 °C ± 2 in a refrigerator until all laboratory analyses were performed.

### 2.4. Measurement of Flour Water Activity and Colour

The water activity of samples was measured using a water activity meter (model LabSwift-aw, Novasina AG, Lachen, Switzerland) at room temperature (25 ± 1 °C). The Commission Internationale de l’Éclairage (CIE) color parameters (L*, a, b*) of flours were measured with a colorimeter (CR-400 Konica Minolta Inc., Marunouchi, Japan) equipped with a DP-400 data processor in accordance with the procedure described by [12]. The chroma meter was calibrated prior to analysis using the manufacturer’s standard white plate at D65 illumination (Y = 80.1, x = 0.3219 and y = 0.3394). Chroma index (C*) and total colour change (∆E*) were calculated from the L*, a* b* values using Equations (1) and (2) [12]:

(1) $$C*=\sqrt{{\left(a^{*}\right)}^{2}+{\left(b^{*}\right)}^{2}}$$

(2) $$∆E*=\sqrt{{\left(L_{0}^{*}-L^{*}\right)}^{2}+{\left(a_{0}^{*}-a^{*}\right)}^{2}+{\left(b_{0}^{*}-b^{*}\right)}^{2}}$$

where $L_{0}^{*},\;a_{0}^{*},\;b_{0\;}^{*}$ represent CIE color parameters of wheat flour and $L^{*},a^{*},b^{*}$ are colour values of OFSP composite flours. Five replicate measurements were taken for each sample.

### 2.5. Determination of Proximate and Mineral Compositions

Proximate compositions (moisture, crude protein, fat, crude fiber and ash) of composite flours were determined using the standard official methods of AOAC [13] and total carbohydrate was calculated by applying the difference method. The Atwater calorie conversion factors method was used to calculate the energy value (kcal/100 g_DM_) of flour [14]. The concentrations of calcium, potassium, magnesium, iron, sodium and zinc were analyzed using an atomic absorption spectrophotometer (model: 211 VGP, Buck Scientific, East Norwalk, CT, USA) in accordance with the procedures of [15]. All analyses were repeated twice.

### 2.6. Determination of ß-carotene, Vitamin A and Ascorbic Acid

ß-carotene content was analyzed using the procedure established by Rodriguez-Amaya and Kimura [16] using petroleum ether for extraction and partitioning of ß-carotene in the samples and absorbance taken at 450 nm with UV/Visible Spectrophotometer (model: C-7000UV, Peak Instruments, Houston, TX, USA). Vitamin A content in flours was calculated by the conversion ratio of 13 μg ß-carotene: 1 μg retinol activity equivalent reported for sweet potato [5].

Ascorbic acid concentration was measured with the 2,6-dichlorophenolindophenol (DIP) method of Albrecht [17] as described by Mohammed at al. [18] Briefly, 5 g of flour was extracted in 5% metaphosphoric acid and titrated against 0.21% DIP dye. The ascorbic acid content measured was expressed as mg/100 g_DM_.

### 2.7. Analysis of Total Phenolic, Flavonoids and Total Antioxidant Activity

#### 2.7.1. Sample Extraction

The procedure introduced by Li et al. [19] was used to extract flour samples with minor modification. In this study, 2 g of flour was dispensed into 16 mL of 80% methanol mixed with 1% HCl and incubated in the dark at room temperature (25 °C ± 2) for 24 h after which the mixture was centrifuged at 4000 rpm for 30 min using Rotofix 32A centrifuge (Andreas Hettich GmbH & Co. KG, Tuttlingen, Germany). The supernatant was collected and the residue was extracted twice. Supernatants collected from three extractions were combined and stored at 4 °C ± 1 until all analyses were done.

#### 2.7.2. Total Phenolic Content

The Folin–Ciocalteu test described by Li et al. [19] was used to analyze total phenolic content in flour extract. Briefly, 0.5 mL of extract or gallic acid standard were mixed with 5 mL of Folin–Ciocalteu reagent (1 mol), followed by an addition of 4 mL of sodium carbonate (7.5%, *w*/*v*). The reaction mixture was incubated for 2 h at room temperature (25 °C ± 2) after which absorbance was taken at 765 nm using a UV/Vis spectrophotometer (Model: C-7000UV, Peak Instruments, Huston, TX, USA). Gallic acid was used to establish standard calibration curve (R^2^ = 0.998) and total phenolic content expressed as mg gallic acid equivalence (mg GAE/100 g_DM_).

#### 2.7.3. Total Flavonoid Content

Total flavonoid content was determined by the colorimetric method [19]. About 0.5 mL of the extract was added to 2 mL of distilled water containing 0.15 mL sodium nitrite (50 g/L). After five minutes, 0.15 mL of 10% AlCl_3_ solution was added and the mixture was kept at room temperature (25 °C ± 2) for 5 min followed by addition of 1 mL of 1 M NaOH. The reaction solution was mixed thoroughly and incubated at room temperature for 15 min after which absorbance was measured at 415 nm with a spectrophotometer. In this study, Catechin was used as standard for the calibration curve (R^2^ = 0.996) and total flavonoid content was expressed as mg Catechin equivalence (mg CE/100 g_DM_).

#### 2.7.4. Analysis of Total Antioxidant Activity

Total antioxidant activity of flour extracts was measured by the phosphomolydenum complex method of Prieto et al. [20] Briefly, 0.1 mL extract was added to 1 mL of reagent solution (0.6 M H_2_SO_4_, 28 mM sodium phosphate and 4 mM ammonium molybdate) and incubated in a water bath (JP Selecta S.A., Barcelona, Spain) at 95 °C for 90 min after which absorbance was measured at 695 nm against the blank (0.1 mL extraction solvent and 1 mL reagent solution). Ascorbic acid was used as standard and total antioxidant activity values expressed as mg ascorbic acid equivalence/100 g_DM_.

### 2.8. Determination of Flour Functional Properties

#### 2.8.1. Loose and Packed Bulk Densities

Loose and tapped bulk densities was measured using the method described by Elkhalifa et al. [21] with some modifications. The flour samples (50 g) were measured using a precision balance (model: PBJ 620-3M, KERN & SOHN GmbH, Balingen, Germany) into a 250 mL measuring cylinder and the volume recorded (V_o_) was followed by gentle tapping on the bench surface from a height of about 10 cm until the volume remained constant. The final volume of flour was measured (V_1_) and bulk densities were calculated using the following Equations:

(3) $$\mathrm{Loose}\;\mathrm{bulk}\;\mathrm{density}\;(g/\mathrm{mL})=\frac{\mathrm{Weight}\;\mathrm{of}\;\mathrm{flour}\;}{\mathrm{Volume}\;\mathrm{of}\;\mathrm{untapped}\;\mathrm{flour}\;(V_{0})}$$

(4) $$\mathrm{Tapped}\;\mathrm{bulk}\;\mathrm{density}\;(g/\mathrm{mL})=\frac{\mathrm{Weight}\;\mathrm{of}\;\mathrm{flour}\;}{\mathrm{Volume}\;\mathrm{of}\;\mathrm{tapped}\;\mathrm{flour}\;(V_{1})}$$

#### 2.8.2. Water Absorption Capacity

Water absorption capacity (WAC) was determined using the procedure described by Awolu [22] with a minor modification. Flour (2 g) was measured into a clean pre-weighed 15 mL centrifuge tube and the weight of the tube with the sample was measured (W_1_). Distilled water (10 mL) was added to the tube and was then vortexed for 1 min and kept at room temperature (25 °C) for 30 min followed by centrifugation at 4000 rpm for 30 min. The supernatant was gently poured into a beaker and free excess water was drained by inverting the tubes on Whatman No. 1 filter paper. The final weight of the centrifuge tube containing the sample after draining water was measured (W_2_) and WAC was expressed as grams of water absorbed per gram of flour as shown below:

(5) $$\mathrm{WAC}\;(\%)=\frac{\mathrm{Amount}\;\mathrm{of}\;\mathrm{water}\;\mathrm{absorbed}\;(W_{2}\;-W_{1})}{\mathrm{Initial}\;\mathrm{sample}\;\mathrm{weight}\;(g)}\;\times 100$$

#### 2.8.3. Oil Absorption Capacity

The protocol used by Elkhalifa et al. [21] was adopted with slight modifications for the determination of oil absorption capacity (OAC) of the flours. One gram of flour (W_1_) was transferred into a clean empty centrifuge tube with known weight (W_2_) and 10 mL of soybean oil added. The mixture was vortexed for 30 s, kept at room temperature (25 °C) for 30 min and centrifuged for 30 min at 4000 rpm. Unabsorbed oil was carefully drained, after which the weight of the tube plus the sample was measured (W_3_) and OAC was calculated using the following expression:

(6) $$\mathrm{OAC}\;(\%)=\frac{\mathrm{Amount}\;\mathrm{of}\;\mathrm{oil}\;\mathrm{absorbed}\;\left(g\right)}{\mathrm{Initial}\;\mathrm{sample}\;\mathrm{weight}\;\left(g\right)}=\frac{W_{3}-\left(W_{1}+W_{2}\right)}{W_{1}}\;\times 100$$

#### 2.8.4. Swelling Capacity and Water Solubility

Swelling capacity and water solubility were measured by the methods described by Olatunde et al. [7] with a few modifications. Briefly, 1 g of flour was added to a weighed 15 mL centrifuge tube (W_1_) and 12.5 mL distilled water was added. The sample mixture in the tube was vortexed for 1 min, heated for 30 min in a water bath at 60 °C with regular stirring at 5 min interval and centrifuged at 4000 rpm for 30 min. Supernatant was transferred into a weighed crucible (W_2_) and the final weight of the tube plus the gel measured (W_3_) and the swelling capacity was calculated using Equation (7). The crucible with the supernatant was dried in an electric oven (JP Selecta S.A, Barcelona, Spain) at 105 °C until the weight remained unchanged. The weight of the crucibles plus the dry solids was measured (W_4_) after cooling in a desiccator and water solubility was calculated using Equation (8).

(7) $$\mathrm{Swelling}\;\mathrm{capacity}\;(\%)=\frac{\mathrm{Weight}\;\mathrm{of}\;\mathrm{gel}\;\mathrm{formed}\;(W_{3}-W_{1})}{Initial\;flour\;weight\;\left(g\right)}\;\times 100$$

(8) $$\mathrm{Water}\;\mathrm{solubility}\;(\%)=\frac{\mathrm{Amount}\;\mathrm{of}\;\mathrm{solids}\;\mathrm{in}\;\mathrm{supernatant}\;(W_{4}-W_{2})}{Initial\;flour\;weight\;\left(g\right)}\;\times 100$$

### 2.9. Analysis of Flour Pasting Properties

A Rapid Visco Analyzer (model RVA 4500, Perten Instruments, Hägersten, Sweden) connected to a personal computer equipped with the manufacturer’s Thermocline for Windows software for operations and data management was used to analyze the pasting profile of the flours using standard procedure [22]. The heating and cooling cycle settings were: slurry (3 g flour and 25 mL distilled water on 14% moisture basis), which was held at 50 °C for 1 min, heated to 95 °C and held at this temperature for 10 min and finally cooled to 50 °C and held for 2 min. Mixing was done at a contact rate (160 rpm) and analysis was repeated twice.

### 2.10. Statistical Analysis

Data obtained was subjected to multivariate analysis in a full factorial design of general linear model analysis of variance using SPSS software (IMB SPSS Statistics, version 25). Where significant difference occurred, means were separated using Tukey pairwise test at 5% significance level.

## 3. Results and Discussion

### 3.1. Physical Properties of Peeled and Unpeeled OFSP Composite Flours

**Figure 2:**
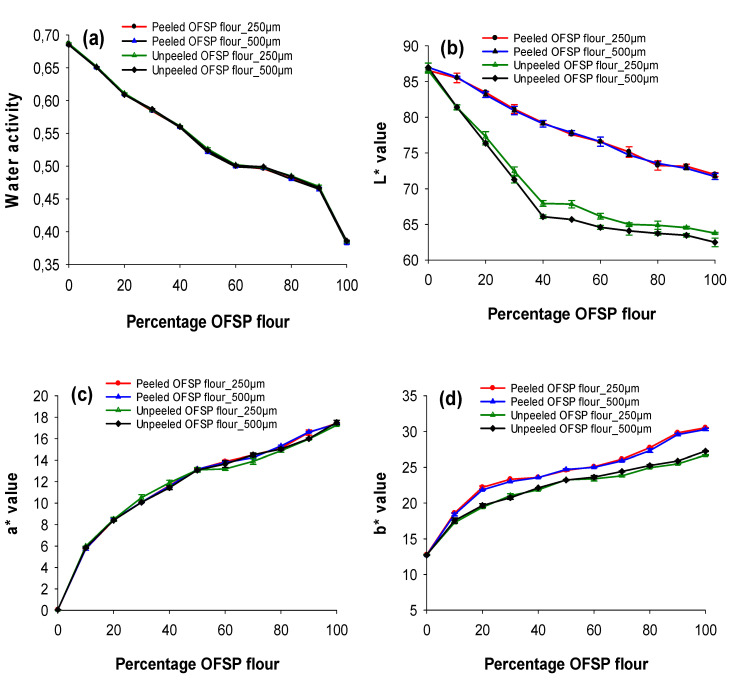

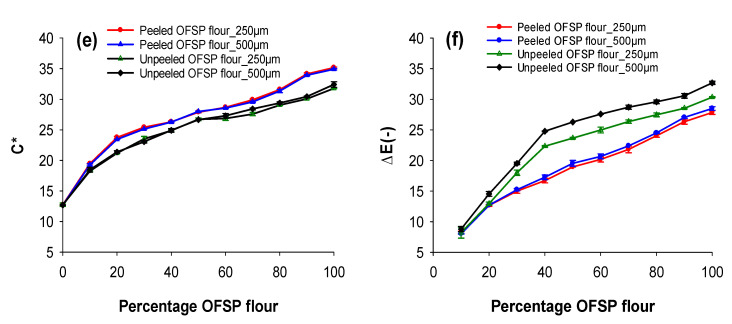
Effect of sieve particle size and blend proportion on the physical properties of peeled and unpeeled OFSP composite flours. (**a**) Water activity; (**b**) L* (lightness); (**c**) a* (redness); (**d**) b* (yellowness); (**e**) C* (Chroma); (**f**) ∆E (−) (total colour difference). Values are expressed as means ± standard deviation (*n* = 5). Significant difference between means was determined at *p* < 0.05.

Figure 2a shows the water activity (a_w_) of OFSP composite flours. It was observed that a_w_ values varied between 0.382 and 0.687. The peeled and unpeeled OFSP flours did not vary significantly (*p* > 0.05) in water activity. Similarly, the effect of sieve particle size on a_w_ was insignificant (*p* > 0.05). Nevertheless, a decreasing tread in a_w_ was detected as OFSP flour levels increased in the composite flours. The low a_w_ of OFSP flours would enhance its stability and shelf life during storage.

The CIE colour attributes of peeled and unpeeled OFSP composite flours varied significantly (*p* < 0.05) and ranged between L* (62.49–86.97), a* (0.05–17.51), b* (12.72–30.50), C* (12.72–35.15) and ∆E (8.04–32.69) as illustrated in Figure 2b–f respectively. The a* and b* values were higher while L* was lower in 100% of the peeled and unpeeled OFSP flours than the values reported for the OFSP flours [3,23]. The peeled OFSP flour had a slightly higher L* and b*, with lower ∆E values than the unpeeled flours. This result agreed with earlier report that unpeeled sweet potato flours were darker than peeled flours [4] but disagreed with Hamed at al. [8], who reported that peeling had no significant effect on the color of sweet potato flours. The 250 μm sieve flours had higher L* values among the unpeeled OFSP composite flours (Figure 2b), but lower ∆E than corresponding 500 μm mesh flours for both peeled and unpeeled composite flours (Figure 2f). The L* value decreased, whereas a*, b*, C* and ∆E* increased as OFSP flour increased in the composite flours. This confirmed the findings of Singh et al. [24], who observed that L* values decreased while b* increased as sweet potato flour levels increased in wheat composite flours.

### 3.2. Proximate Composition and Energy Value of Peeled and Unpeeled OFSP Composite Flours

The proximate composition and energy value of the peeled and unpeeled OFSP composite flours varied between 5.27–11.52, 6.13–12.78, 0.70–1.62, 0.54–2.65, 1.10–3.40, 72.43–81.60 g/100 g_DM_ and 355.45–362.58 kcal/100 g for moisture, crude protein (CP), fat, crude fiber (CF), ash, total carbohydrate and energy value respectively (Table 1). The effect of sieve particle size on the proximate composition of peeled and unpeeled OFSP composite flours was insignificant (*p* > 0.05). It was observed that peeled OFSP-based flours had slightly lower CF and total carbohydrate but higher CP than the unpeeled OFSP composite flours. In addition, the proximate values of OFSP composite flours showed decreasing trends for moisture, CP and fat, whereas CF, ash and total carbohydrate increased as wheat flour levels decreased (Table 1). The CP and fat contents of 100% peeled and unpeeled flours were within the values reported for OFSP flours by Fana et al. [25], whereas the CP and fat were higher and lower respectively than the values stated by Kuyu et al. [26] Similarly, the moisture, ash and carbohydrate content of 100% OFSP flours were all within the values reported by other authors [25,26]. The CF and energy value of both peeled and unpeeled OFSP flours agreed with the findings of Fana et al. [25] and Rodrigues et al. [27], but higher than the values reported by Kuyu et al. [26]

### 3.3. Mineral Composition of Peeled and Unpeeled OFSP Composite Flours

The concentrations of Ca, K, Mg, Fe, Na and Zn measured in peeled and unpeeled OFSP composite flours varied significantly (*p* < 0.05) between 6.47–22.80, 71.67–757.88, 4.52–6.64, 2.78–4.24, 3.72–4.46 and 1.32–2.36 mg/100 g_DM_ as presented in Table 2. The sieve particle size did not significantly (*p* > 0.05) influence the mineral levels measured. In addition, with the exception of Ca and K that differed among the peeled and unpeeled OFSP flours, the remaining minerals did not vary significantly between the respective peeled and unpeeled OFSP composite flours. There was a significant (*p* < 0.05) increase in Ca, K, Mg and Fe, whereas Na and Zn decreased as the proportions of OFSP flours increased (Table 2). Among the minerals measured in both peeled and unpeeled OFSP composite flours, K was the predominant, followed by Ca, while the least was Zn. The Fe concentration in 100% peeled OFSP flour was similar to the value reported by Tumuhimbise et al. [28]

### 3.4. Bioactive Compounds and Total Antioxidant Activity in Peeled and Unpeeled OFSP Composite Flours

The concentrations (per 100 g_DM_) of ß-carotene, vitamin A, ascorbic acid, total phenolic content, total flavonoid content and total antioxidant activity observed in the peeled and unpeeled OFSP composite flours varied greatly (*p* < 0.001) between 3.5–9530.0 μg, 0.27–733.08 μg retinol activity equivalent (RAE), 5.90–35.72 mg, 36.76–186.50 mg gallic acid equivalent (GAE), 29.00–81.70 mg Catechin and 106.79–335.82 mg ascorbic acid equivalent (AAE) respectively (Table 3). The unpeeled OFSP composite flours had a significantly (*p* < 0.05) higher ß-carotene, vitamin A, total phenolic, total flavonoid and total antioxidant activity values but lower ascorbic acid content than the corresponding peeled OFSP composite flours. This could be attributed to the high phenolics and flavonoid concentrations in the peels rather than the flesh of the potato [29]. The effect of sieve particle size on the bioactive compounds and total antioxidant activity was insignificant (*p* > 0.05). However, the levels of these compounds in the composite flours increased as the proportion of OFSP flour increased. The ß-carotene values measured in the 100% peeled and unpeeled OFSP flours were higher than the values reported for OFSP flours [3], but were within the range of values stated by Fana et al. [25] In addition, the total phenolic contents of 100% peeled and unpeeled OFSP flours were higher than the values reported by Kuyu et al. [26]

### 3.5. Functional Properties of Peeled and Unpeeled OFSP Composite Flours

**Figure 3:**
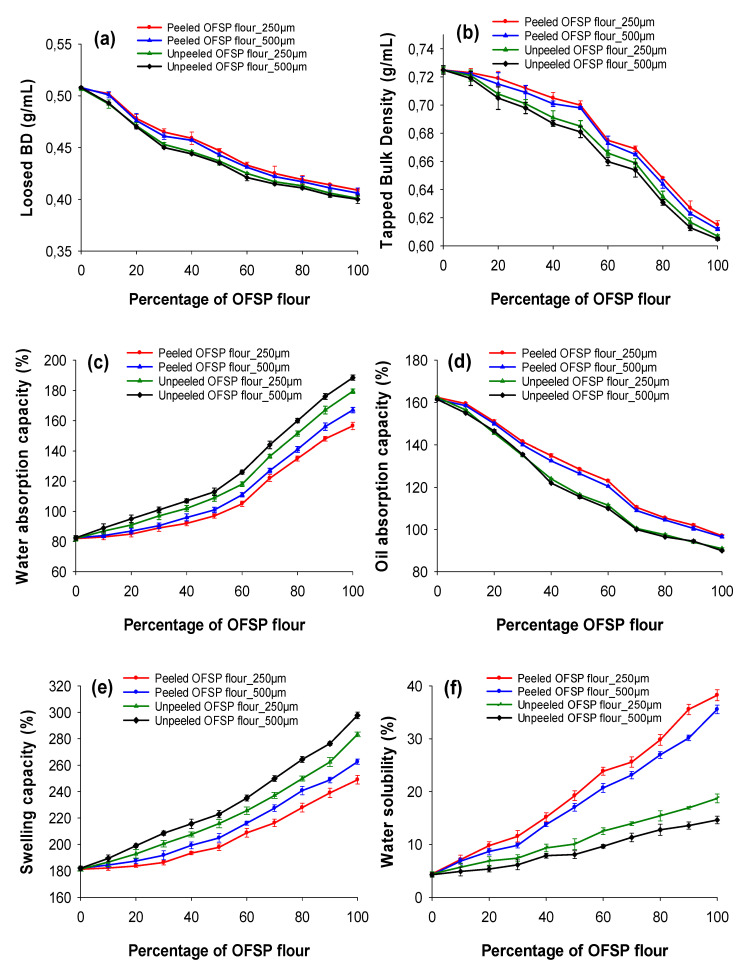
Effect of sieve particle size and blend proportions on the functional properties of peeled and unpeeled OFSP composite flours. (**a**) Loose bulk density (BD); (**b**) Tapped BD; (**c**) Water absorption capacity; (**d**) Oil absorption capacity; (**e**) Swelling capacity; (**f**) Water solubility. Values are expressed as mean ± standard deviation (*n* = 2). Significant difference between means was determined at *p* < 0.05.

Functional properties of flour determines the direct use of flour or its application in food processing [7]. The functional properties of peeled and unpeeled OFSP composite flours are shown in Figure 3. The loose and tapped bulk density of the OFSP composite flours varied between 0.400–0.508 g/mL and 0.605–0.725 g/mL respectively. The tapped bulk density of flour measurements were consistent with the results reported for OFSP flours by Tumuhimbise et al. [28], but lower than that values reported by Fana et al. [25] In general, the low bulk density of OFSP flours make them a more suitable ingredient for baby food [22].

WAC describes the ability of flour to absorb water and swell, which is an important determinant of product yield and consistency [28]. The unpeeled OFSP flour had a significantly (*p* < 0.05) higher WAC (179.5–188.5%) as compared to peeled OFSP flour (156.5–167.0%) and wheat flour which had the lowest levels (82.3 ± 1.51%). The WAC of the 100% OFSP flours were lower than the values mentioned by Fana et al. [25]

The swelling capacity of OFSP composite flours varied between 181.2–297.6%. The unpeeled OFSP flours had higher WAC and swelling capacity than the peeled OFSP flours and this can be attributed to their differences in chemical composition [30]. The OFSP flours sieved with 250 μm mesh size had a significantly (*p* < 0.05) lower WAC and swelling capacity than their corresponding 500 μm mesh size flours (Figure 3c,d respectively). These results confirm the earlier observation that WAC of reconstituted whole wheat flour reduces as particle size decreases [9]. The WAC of the composite flours increased with the increased proportion of OFSP flours, and this was in line with the findings of Singh et al. [24]

OAC is an important property of flour since fats retain flavors and improve the mouth feel and palatability of bakery products [31]. The OAC of the OFSP flours ranged between 90.0–97.0% and was within the range of values measured by Fana et al. [25], but higher than the values measured by Rodrigues et al. [27] The effect of sieve particle size on OAC of OFSP composite flours was insignificant (*p* > 0.05), but OAC decreased as OFSP addition increased (Figure 3e) and this could be related to the variation in chemical composition such as the low protein content of OFSP flour [30].

The water solubility was significantly (*p* < 0.001) higher in the peeled OFSP composite flours (6.86–38.26%) than the values of the unpeeled OFSP composite flours (4.91–14.64%) as shown in Figure 3f. The 250 μm sieved composite flours recorded a slightly higher water solubility than their corresponding 500 μm mesh flours. The water solubility of the composite flours increased as the OFSP flour proportion increased and this could be attributed to the high sugar content of OFSP flour [32]. The water solubility of the 100% peeled OFSP flour was similar, while that of the unpeeled OFSP flours was lower than the values reported by Rodrigues et al. [27]

### 3.6. Pasting Properties of Peeled and Unpeeled OFSP Composite Flours

The peak, trough, breakdown, final, setback viscosities, peak time and pasting temperature of 100% peeled and unpeeled OFSP flours measured ranged between 96.5–108.5, 33.5–36.0, 63.0–72.5, 50.0–52.0 and 15.0–17.5 centipoise (cP); 4.12–4.15 min and 83.90–83.98 °C respectively were significantly (*p* < 0.001) lower than those of the viscosities of 100% wheat flour (Table 4). The peeled and unpeeled OFSP flours only differed slightly in the peak and breakdown viscosities where the unpeeled OFSP flours had slightly higher values. The effect of sieve particle size on the pasting properties of OFSP composite flours was insignificant (*p* > 0.05).

Generally, the pasting properties of composite flours decreased as the OFSP flour proportions increased. This could be best explained by differences in the chemical compositions of the flours [30]. Apart from breakdown viscosity, the 100% peeled and unpeeled OFSP flours had lower viscosities than the values stated by Ruttarattanamongkol et al. [3] On the contrary, peak and breakdown viscosities of the peeled and unpeeled OFSP flours measured were higher while trough and final viscosities were similar to values measured in blanched OFSP flours [23]. This can be linked to the variations in chemical compositions of the composite flours [30]. The lower final viscosity of OFSP flours indicates a decreased thickness of paste when cooled to 50 °C.

Retrogradation increases starch resistance to enzymatic hydrolysis, increases the staling rate of bread and affects the stability of other starchy food products [33]. Therefore, the peeled and unpeeled OFSP flours would be suitable in the preparation of infant foods due to their low setback viscosity [28] and could be suitable for cookie development due to their low viscosity. Peak time and pasting temperature obtained for 100% peeled and unpeeled OFSP flours were lower than the values of blanched OFSP flours reported by Jangchud et al. [23] The lower peak time and pasting temperature of OFSP flours suggest a shorter cooking time and a lower energy consumption during cooking as compared to wheat flour.

## 4. Conclusions

This research has showed that peeling significantly reduces crude fiber, total carbohydrate, ß-carotene, vitamin A, total phenolic content, total flavonoid content, total antioxidant activity, water absorption capacity and swelling capacity but increases crude protein, oil absorption capacity and lightness (L*) of OFSP flours. The effect of sieve particle size on nutritional composition and pasting properties was insignificant. Nevertheless, OFSP flour sieved with a 500 μm mesh size had a significantly higher water absorption capacity and swelling capacity but a lower water solubility than 250 μm mesh flours in our study. In addition, OFSP flours had higher crude fiber, ash, total carbohydrate and bioactive compounds, but lower crude protein and pasting properties when compared to wheat flour. Moreover, the proportion of OFSP flour greatly affected all the quality properties of peeled and unpeeled OFSP–wheat composite flours. The results showed that unpeeled and peeled OFSP flour blends sieved with a 250 μm or 500 μm mesh size have great potentials for an application in the food industry, mainly in the manufacturing of products like biscuits, cookies, breads, noodles and baby foods. This study recommends further investigation into the effects of peeling, sieve particle size and flour blends on the quality properties of food products such as bakery foods.

## Figures and Tables

**Table 1:** Effect of sieve particle size and blend proportion on proximate composition and energy value of peeled and unpeeled OFSP composite flours.

OFSP Flour Processing	Wheat: OFSP Flour (%)	Proximate Composition (g/100 g_DM_)						
		Moisture	Crude Protein	Fat	Crude Fiber	Ash	Carbohydrates	Energy (Kcal/100 g)
Peeled _ 250 μm sieve particle size	100:0	11.52 ± 0.00^a^	12.79 ± 0.03^a^	1.62 ± 0.01^a^	0.54 ± 0.03^s^	1.10 ± 0.01^q^	72.43 ± 0.24^q^	355.42 ± 0.78^op^
	90:10	10.76 ± 0.03^b^	12.43 ± 0.09^ab^	1.49 ± 0.03^abc^	0.71 ± 0.03^rs^	1.19 ± 0.03^opq^	73.42 ± 0.17^p^	356.77 ± 0.25^klmnop^
	80:20	10.14 ± 0.01^c^	11.98 ± 0.13^c^	1.37 ± 0.01^cde^	0.77 ± 0.01^pqr^	1.32 ± 0.01^mnopq^	74.42 ± 0.15^no^	357.87 ± 0.40^fghijklmno^
	70:30	9.46 ± 0.01^de^	11.23 ± 0.05^ef^	1.29 ± 0.01^cdefg^	0.86 ± 0.01^pqr^	1.74 ± 0.01^klm^	75.43 ± 0.20^lm^	358.23 ± 0.23^efghijklm^
	60:40	8.92 ± 0.00^fg^	10.85 ± 0.01^fgh^	1.16 ± 0.04^efghij^	0.93 ± 0.03^opq^	1.90 ± 0.00^ijkl^	76.25 ± 0.03^jk^	358.78 ± 0.37^defghijkl^
	50:50	8.61 ± 0.03^fg^	10.39 ± 0.08^j^	1.09 ± 0.03^ghijkl^	1.10 ± 0.01^mno^	2.26 ± 0.01^ghi^	76.55 ± 0.08^jk^	357.57 ± 0.25^fghijklmnop^
	40:60	7.02 ± 0.02^hi^	9.77 ± 0.05^kl^	1.03 ± 0.03^ijklmn^	1.32 ± 0.04^jkl^	2.50 ± 0.04^efgh^	78.38 ± 0.01^efg^	361.83 ± 0.08^ab^
	30:70	6.18 ± 0.02^jkl^	9.42 ± 0.04^klm^	0.98 ± 0.01^jklmnop^	1.66 ± 0.06^gh^	2.75 ± 0.01^cdef^	79.02 ± 0.41^de^	362.58 ± 1.68^a^
	20:80	6.12 ± 0.00^klm^	8.65 ± 0.01^n^	0.95 ± 0.02^jklmnopq^	1.99 ± 0.01^f^	3.00 ± 0.03^abcd^	79.31 ± 0.17^cd^	360.33 ± 0.46^abcde^
	10:90	5.42 ± 0.02^n^	7.93 ± 0.04^o^	0.91 ± 0.01^lmnopqrs^	2.18 ± 0.03^de^	3.23 ± 0.01^ab^	80.35 ± 0.06^b^	361.25 ± 0.12^abc^
	0:100	5.27 ± 0.02^n^	7.54 ± 0.01^opq^	0.87 ± 0.01^mnopqrs^	2.45 ± 0.05^bc^	3.40 ± 0.00^a^	80.47 ± 0.02^b^	359.89 ± 0.29^bcdefg^
Peeled _ 500 μm sieve particle size	100:0	11.51 ± 0.03^a^	12.77 ± 0.11^ab^	1.60 ± 0.01^ab^	0.54 ± 0.01^s^	1.10 ± 0.04^q^	72.48 ± 0.37^q^	355.40 ± 0.25^p^
	90:10	10.84 ± 0.01^b^	12.39 ± 0.01^b^	1.48 ± 0.01^abc^	0.72 ± 0.01^rs^	1.18 ± 0.03^opq^	73.41 ± 0.08^p^	356.48 ± 0.24^lmnop^
	80:20	10.21 ± 0.01^c^	11.94 ± 0.01^cd^	1.35 ± 0.03^cdef^	0.79 ± 0.01^pqr^	1.31 ± 0.01^nopq^	74.42 ± 0.13^no^	357.57 ± 0.68^fghijklmnop^
	70:30	9.55 ± 0.00^d^	11.19 ± 0.06^efg^	1.26 ± 0.02^defgh^	0.89 ± 0.04^pqr^	1.72 ± 0.07^klmn^	75.41 ± 0.02^lm^	357.66 ± 0.15^fghijklmnop^
	60:40	9.00 ± 0.01^efg^	10.81 ± 0.01^ghi^	1.14 ± 0.04^fghijk^	0.95 ± 0.02^nop^	1.88 ± 0.03^ijkl^	76.24 ± 0.14^jk^	358.4 ± 0.16^defghijklm^
	50:50	8.59 ± 0.01^g^	10.34 ± 0.03^j^	1.05 ± 0.01^hijklmn^	1.15 ± 0.04^lm^	2.25 ± 0.06^hij^	76.63 ± 0.11^ijk^	357.31 ± 0.41^hijklmnop^
	40:60	7.26 ± 0.01^h^	9.73 ± 0.04^kl^	1.01 ± 0.02^jklmno^	1.35 ± 0.06^ijk^	2.47 ± 0.03^fgh^	78.20 ± 0.07^fg^	360.73 ± 0.37^abcd^
	30:70	7.00 ± 0.01^hi^	9.40 ± 0.11^lm^	0.95 ± 0.01^jklmnopq^	1.68 ± 0.04^g^	2.73 ± 0.01^cdef^	78.25 ± 0.03^efg^	359.15 ± 0.55^cdefghijk^
	20:80	6.61 ± 0.00^ij^	8.63 ± 0.06^n^	0.93 ± 0.01^klmnopqr^	2.01 ± 0.03^ef^	2.99 ± 0.02^abcd^	78.84 ± 0.06^def^	358.21 ± 0.36^efghijklm^
	10:90	5.90 ± 0.05^lm^	7.88 ± 0.04^op^	0.89 ± 0.01^lmnopqrs^	2.20 ± 0.01^d^	3.20 ± 0.01^ab^	79.94 ± 0.23^bc^	359.27 ± 0.22^cdefghij^
	0:100	5.28 ± 0.02^n^	7.51 ± 0.01^pq^	0.85 ± 0.03^nopqrs^	2.49 ± 0.06^abc^	3.37 ± 0.01^a^	80.51 ± 0.06^b^	359.71 ± 0.28^bcdefgh^
Unpeeled_ 250 μm sieve particle size	90:10	10.88 ± 0.01^b^	11.94 ± 0.04^cd^	1.38 ± 0.04^bcd^	0.74 ± 0.01^r^	1.13 ± 0.01^q^	73.95 ± 0.06^op^	355.94 ± 0.24^mnop^
	80:20	10.19 ± 0.03^c^	11.56 ± 0.13^de^	1.26 ± 0.02^defgh^	0.81 ± 0.02^pqr^	1.21 ± 0.03^opq^	74.98 ± 0.03^mn^	357.46 ± 0.19^ghijklmnop^
	70:30	9.66 ± 0.06^d^	10.71 ± 0.03^hij^	1.10 ± 0.01^ghijkl^	0.93 ± 0.03^opq^	1.55 ± 0.01^lmnop^	76.06 ± 0.12^kl^	356.92 ± 0.43^ijklmnop^
	60:40	9.07 ± 0.03^ef^	10.43 ± 0.06^ij^	1.04 ± 0.04^hijklmn^	1.09 ± 0.06^mno^	1.80 ± 0.04^kl^	76.59 ± 0.26^ijk^	357.36 ± 1.11^hijklmnop^
	50:50	8.59 ± 0.01^g^	9.80 ± 0.09^k^	0.99 ± 0.02^jklmnop^	1.2 ± 0.01^klm^	2.10 ± 0.00^hijk^	77.35 ± 0.09^hi^	357.43 ± 0.54^hijklmnop^
	40:60	7.36 ± 0.00^h^	9.26 ± 0.08^m^	0.93 ± 0.04^klmnopqr^	1.48 ± 0.01^hij^	2.34 ± 0.02^fgh^	78.64 ± 0.32^def^	359.93 ± 0.58^bcdef^
	30:70	7.04 ± 0.00^hi^	8.63 ± 0.11^n^	0.86 ± 0.01^mnopqrs^	1.79 ± 0.02^g^	2.67 ± 0.04^defg^	79.02 ± 0.07^de^	358.32 ± 0.07^defghijklm^
	20:80	6.62 ± 0.00^ij^	7.48 ± 0.23^q^	0.81 ± 0.01^opqrs^	2.15 ± 0.05^def^	2.89 ± 0.03^bcde^	80.06 ± 0.18^bc^	357.39 ± 0.64^hijklmnop^
	10:90	6.47 ± 0.00^jk^	6.73 ± 0.25^r^	0.77 ± 0.03^pqrs^	2.38 ± 0.01^c^	3.11 ± 0.01^abc^	80.55 ± 0.15^b^	356.01 ± 0.14^mnop^
	0:100	5.67 ± 0.01^mn^	6.15 ± 0.21^s^	0.72 ± 0.02^rs^	2.61 ± 0.01^ab^	3.25 ± 0.00^ab^	81.60 ± 0.27^a^	357.48 ± 0.04^fghijklmnop^
Unpeeled_ 500 μm sieve particle size	90:10	10.86 ± 0.01^b^	11.91 ± 0.01^cd^	1.35 ± 0.01^cdef^	0.76 ± 0.01^qr^	1.17 ± 0.01^pq^	73.97 ± 0.18^op^	355.63 ± 0.85^nop^
	80:20	10.21 ± 0.03^c^	11.52 ± 0.08^e^	1.23 ± 0.01^defghi^	0.81 ± 0.03^pqr^	1.25 ± 0.03^opq^	74.99 ± 0.02^mn^	357.07 ± 0.54^ijklmnop^
	70:30	9.60 ± 0.02^d^	10.69 ± 0.06^hij^	1.08 ± 0.00^ghijklm^	0.95 ± 0.01^nop^	1.60 ± 0.02^lmno^	76.10 ± 0.09^jkl^	356.84 ± 0.39^jklmnop^
	60:40	9.04 ± 0.01^efg^	10.40 ± 0.02^j^	1.01 ± 0.03^ijklmno^	1.12 ± 0.03^mn^	1.83 ± 0.00^jkl^	76.85 ± 0.01^hij^	358.05 ± 0.14^efghijklmn^
	50:50	8.57 ± 0.00^g^	9.78 ± 0.03^kl^	0.97 ± 0.04^jklmnopq^	1.23 ± 0.05^klm^	2.13 ± 0.04^hijk^	77.63 ± 0.11^gh^	358.35 ± 0.07^defghijklm^
	40:60	7.31 ± 0.03^h^	9.22 ± 0.22^m^	0.89 ± 0.03^lmnopqrs^	1.50 ± 0.02^hi^	2.37 ± 0.01^fgh^	78.97 ± 0.28^def^	360.73 ± 0.03^abcd^
	30:70	7.05 ± 0.03^hi^	8.59 ± 0.10^n^	0.84 ± 0.01^nopqrs^	1.81 ± 0.05^g^	2.71 ± 0.01^cdef^	79.36 ± 0.05^cd^	359.30 ± 0.02^cdefghi^
	20:80	6.61 ± 0.03^ij^	7.41 ± 0.02^q^	0.79 ± 0.00^opqrs^	2.19 ± 0.02^de^	2.91 ± 0.02^bcde^	80.21 ± 0.14^b^	357.57 ± 0.48^fghijklmnop^
	10:90	6.42 ± 0.02^jk^	6.70 ± 0.04^r^	0.75 ± 0.03^qrs^	2.43 ± 0.05^c^	3.18 ± 0.01^ab^	80.54 ± 0.06^b^	355.67 ± 0.03^nop^
	0:100	5.65 ± 0.00^mn^	6.13 ± 0.02^s^	0.70 ± 0.01^s^	2.64 ± 0.00^a^	3.27 ± 0.01^ab^	81.61 ± 0.02^a^	357.22 ± 0.13^ijklmnop^

Values in the same column having no superscript letter in common are significantly different at (*p* < 0.05). Values are expressed as mean ± standard deviation (*n* = 2).

**Table 2:** Effect of sieve particle size and blend proportion on mineral composition of peeled and unpeeled OFSP composite flours.

OFSP Flour Processing	Wheat: OFSP Flour (%)	Mineral Composition (mg/100 g_DM_)					
		Ca	K	Mg	Fe	Na	Zn
Peeled _ 250 μm sieve particle size	100:0	6.47^s^	71.67^u^	4.52^n^	2.78^t^	4.46^a^	2.34^a^
	90:10	7.94^r^	141.42^s^	4.80^mn^	2.96^r^	4.40^b^	1.63^jklmn^
	80:20	9.34^p^	207.92^q^	4.99^jklm^	3.15^p^	4.31^cd^	1.55^mno^
	70:30	10.79^n^	280.60^o^	5.20^ijklm^	3.38^n^	4.23^f^	1.43^opq^
	60:40	12.26^l^	346.72^m^	5.37^ij^	3.44^lm^	4.11^gh^	1.72^ijk^
	50:50	13.61^j^	413.87^k^	5.59^fghi^	3.61^j^	4.03^jkl^	1.89^deg^
	40:60	15.03^h^	482.21^i^	5.84^efgh^	3.78^h^	3.98^mno^	1.94^d^
	30:70	16.50^f^	552.56^g^	5.92^cdef^	3.85^fg^	3.90^qrs^	1.50^nop^
	20:80	17.89^e^	623.04^e^	6.20^abcde^	3.97^c^	3.82^vw^	1.26^rst^
	10:90	19.35^d^	689.66^c^	6.37^abc^	4.14^b^	3.79^wxy^	1.58^klmn^
	0:100	20.96^c^	757.96^a^	6.58^a^	4.24^a^	3.72^z^	1.32^qrst^
Peeled _ 500 μm sieve particle size	100:0	6.47^s^	71.70^u^	4.52^n^	2.78^t^	4.46^a^	2.36^a^
	90:10	7.96^r^	141.35^s^	4.80^mn^	2.98^r^	4.40^b^	1.70^ijkl^
	80:20	9.35^p^	207.87^q^	5.00^jklm^	3.17^p^	4.32^cd^	1.67^ijklm^
	70:30	10.81^n^	280.57^o^	5.240^ijklm^	3.41^mn^	4.24^ef^	1.58^klmn^
	60:40	12.28^l^	346.59^m^	5.40^hij^	3.48^kl^	4.13^g^	1.77^ghij^
	50:50	13.67^j^	413.85^k^	5.61^fghi^	3.63^ij^	4.05^ijk^	1.64^jklmn^
	40:60	15.07^h^	482.19^i^	6.35^abcd^	3.87^ef^	3.99^lmn^	1.35^qrst^
	30:70	16.54^f^	552.45^g^	5.98^bcdef^	3.92^cde^	3.92^pqr^	1.90^deg^
	20:80	17.91^e^	623.02^e^	6.22^abcde^	4.04^b^	3.85^tuw^	1.28^rst^
	10:90	19.38^d^	689.54^c^	6.38^abc^	4.18^b^	3.80^wx^	1.56^lmno^
	0:100	20.94^c^	757.94^a^	6.61^a^	4.26^a^	3.74^z^	1.39^pqr^
Unpeeled_ 250 μm sieve particle size	90:10	8.16^q^	140.84^t^	4.82^lmn^	2.90^s^	4.42^ab^	2.15^b^
	80:20	9.76^o^	205.63^r^	5.02^jklm^	3.06^q^	4.33^c^	2.00^cd^
	70:30	11.39^m^	279.45^p^	5.28^ijkl^	3.18^p^	4.28^de^	1.88^degh^
	60:40	13.09^k^	343.20^n^	5.41^hij^	3.27^o^	4.14^g^	1.87^degh^
	50:50	14.75^i^	410.36^l^	5.60^fghi^	3.39^mn^	4.06^ij^	1.24^st^
	40:60	16.32^g^	479.55^j^	5.86^efgh^	3.50^k^	4.00^lm^	1.57^lmno^
	30:70	17.87^e^	551.82^h^	5.92^cdef^	3.59^j^	3.94^opq^	1.35^qrst^
	20:80	19.42^d^	618.78^f^	6.22^abcde^	3.66^i^	3.86^stu^	1.79^eghi^
	10:90	21.25^b^	673.21^d^	6.38^abc^	3.81^gh^	3.80^wx^	1.28^rst^
	0:100	22.78^a^	756.76^b^	6.63^a^	3.92^de^	3.75^yz^	1.33^qrst^
Unpeeled_ 500 μm sieve particle size	90:10	8.17^q^	140.82^t^	4.83^klmn^	2.91^s^	4.41^b^	2.13^bc^
	80:20	9.78^o^	205.54^r^	5.03^jklm^	3.08^q^	4.34^c^	1.92^de^
	70:30	11.41^m^	279.40^p^	5.30^ijk^	3.19^p^	4.28^de^	1.74^hij^
	60:40	13.12^k^	343.10^n^	5.44^ghij^	3.29^o^	4.13^g^	1.70^ijkl^
	50:50	14.77^i^	410.33^l^	5.62^fghi^	3.40^mn^	4.08^hi^	1.97^d^
	40:60	16.34^g^	479.52^j^	5.88^defg^	3.52^k^	4.01^klm^	1.59^klmn^
	30:70	17.92^e^	551.76^h^	5.97^bcdef^	3.61^j^	3.95^nop^	1.21^t^
	20:80	19.45^d^	618.67^f^	6.27^abcde^	3.68^i^	3.88^rst^	1.64^jklmn^
	10:90	21.27^b^	673.16^d^	6.41^ab^	3.82^gh^	3.82^uvw^	1.29^qrst^
	0:100	22.80^a^	756.75^b^	6.64a	3.93^cd^	3.76^xyz^	1.37^pqrs^

Means in the same column having no superscript letter in common are significantly different at (*p* < 0.05). Values represent means of two replicate measurements.

**Table 3:** Effect of sieve particle size and blend proportion on the bioactive compounds of peeled and unpeeled OFSP composite flours.

OFSP Flour Processing	Wheat: OFSP Flour (%)	Bioactive Compounds and TAA (per 100 g_DM_)					
		Beta-Carotene (µg)	Vitamin A (µg RAE)	Ascorbic Acid (mg)	TPC (mg GAE)	TFC (mg CE)	TAA (mg AAE)
Peeled _250 μm sieve particle size	100:0	3.5 ± 0.71^u^	0.27 ± 0.01^u^	5.90 ± 0.42^r^	36.76 ± 0.62^p^	29.00 ± 0.18^t^	106.83 ± 1.47^t^
	90:10	1087.6 ± 5.66^t^	83.62 ± 0.43^t^	8.87 ± 0.37^q^	47.82 ± 0.23^o^	40.72 ± 0.74^s^	114.68 ± 1.91^st^
	80:20	1969.0 ± 5.56^r^	151.47 ± 1.05^r^	12.01 ± 0.18^op^	56.51 ± 0.40^mn^	41.69 ± 0.18^s^	127.03 ± 1.66^qr^
	70:30	2507.5 ± 5.66^p^	192.85 ± 0.72^p^	14.69 ± 0.33^n^	69.06 ± 0.21^jkl^	45.54 ± 0.22^qr^	154.85 ± 1.75^o^
	60:40	3248.0 ± 1.31^n^	249.85 ± 0.86^n^	18.86 ± 0.48^jkl^	76.88 ± 0.55^i^	49.17 ± 0.18^nop^	167.08 ± 1.23^n^
	50:50	3631.1 ± 8.38^m^	279.31 ± 1.50^m^	20.95 ± 0.27^hij^	85.62 ± 0.38^h^	53.38 ± 0.90^m^	197.10 ± 1.96^k^
	40:60	4417.4 ± 4.24^k^	339.77 ± 0.34^k^	23.89 ± 0.25^fg^	93.79 ± 0.51^g^	57.05 ± 0.52^kl^	214.65 ± 2.74^j^
	30:70	5078.0 ± 8.49^i^	390.62 ± 0.69^i^	26.15 ± 0.59^e^	106.36 ± 0.84^f^	64.11 ± 0.34^ij^	231.33 ± 1.05^i^
	20:80	5728.7 ± 9.50^g^	440.62 ± 1.84^g^	29.63 ± 0.17^c^	121.81 ± 0.26^e^	70.20 ± 0.19^fg^	243.07 ± 1.96^h^
	10:90	6359.0 ± 5.36^f^	489.16 ± 2.72^f^	32.60 ± 0.51^b^	142.50 ± 0.92^c^	74.64 ± 0.37^de^	270.62 ± 1.16^d^
	0:100	6910.5 ± 4.24^d^	531.54 ± 0.58^d^	35.72 ± 0.66^a^	155.00 ± 0.79^b^	79.50 ± 0.58^bc^	322.58 ± 1.63^b^
Peeled _500 μm sieve particle size	100:0	3.5 ± 0.71^u^	0.27 ± 0.01^u^	5.86 ± 0.19^r^	36.42 ± 0.27^p^	29.16 ± 0.22^t^	106.79 ± 1.58^t^
	90:10	1090.0 ± 2.83^t^	83.85 ± 0.25^t^	8.53 ± 0.25^q^	47.96 ± 0.66^o^	40.77 ± 0.19^s^	115.46 ± 1.58^st^
	80:20	1971.3 ± 4.24^r^	151.62 ± 0.33^r^	11.97 ± 0.38^op^	57.15 ± 0.24^m^	41.85 ± 0.24^s^	127.90 ± 2.07^qr^
	70:30	2513.9 ± 5.66^p^	193.31 ± 0.44^p^	14.38 ± 0.24^n^	69.44 ± 0.213^jk^	46.12 ± 0.33^pqr^	155.83 ± 1.20^o^
	60:40	3255.0 ± 1.80^n^	250.39 ± 1.52^n^	18.52 ± 0.16^l^	77.29 ± 0.43^i^	49.51 ± 0.29^no^	168.25 ± 1.81^n^
	50:50	3645.1 ± 5.46^m^	280.39 ± 1.96^m^	20.74 ± 0.51^ijk^	86.13 ± 0.37^h^	53.86 ± 0.47^lm^	198.48 ± 1.41^k^
	40:60	4421.5 ± 9.90^k^	340.08 ± 0.76^k^	23.66 ± 0.55^g^	94.50 ± 0.48^g^	57.40 ± 0.53^k^	215.51 ± 1.99^j^
	30:70	5093.0 ± 8.38^i^	391.77 ± 1.08^i^	26.08 ± 0.44^e^	107.10 ± 0.95^f^	64.55 ± 0.71^hi^	232.95 ± 2.05^i^
	20:80	5744.8 ± 7.07^g^	441.85 ± 0.54^g^	29.59 ± 0.77^c^	122.73 ± 0.67^e^	70.46 ± 0.26^fg^	244.78 ± 1.54^gh^
	10:90	6365.3 ± 4.31^f^	489.62 ± 0.87^f^	32.45 ± 0.38^b^	143.25 ± 0.29^c^	75.19 ± 0.43^de^	272.56 ± 1.19^d^
	0:100	6925.9 ± 2.63^d^	532.69 ± 1.74^d^	35.60 ± 0.64^a^	154.08 ± 0.36^b^	79.63 ± 0.37^bc^	323.70 ± 1.27^b^
Unpeeled _250 μm sieve particle size	90:10	1179.8 ± 5.16^s^	90.75 ± 0.47^s^	6.34 ± 0.27^r^	51.03 ± 0.25^o^	41.23 ± 0.24^s^	123.05 ± 1.65^rs^
	80:20	2307.5 ± 4.24^q^	177.46 ± 0.30^q^	8.71 ± 0.16^q^	63.79 ± 0.37^l^	43.59 ± 0.53^rs^	135.72 ± 1.87^pq^
	70:30	3152.5 ± 3.54^o^	242.50 ± 0.27^o^	10.65 ± 0.49^pq^	73.41 ± 0.53^ij^	47.27 ± 0.47^opq^	165.50 ± 1.75^n^
	60:40	3788.4 ± 8.49^l^	291.39 ± 0.66^l^	13.68 ± 0.25^no^	85.67 ± 0.34^h^	51.78 ± 0.23^mn^	179.85 ± 1.10^lm^
	50:50	4871.5 ± 6.97^j^	374.70 ± 1.31^j^	15.19 ± 0.11^mn^	93.51 ± 0.22^g^	56.91 ± 0.19^kl^	211.47 ± 2.68^j^
	40:60	5546.0 ± 2.53^h^	426.62 ± 2.55^h^	17.37 ± 0.27^l^	103.49 ± 0.73^f^	61.25 ± 0.62^j^	229.82 ± 1.64^i^
	30:70	6793.1 ± 6.87^e^	522.54 ± 2.06^e^	18.96 ± 0.18^jkl^	119.88 ± 0.58^e^	67.62 ± 0.17^gh^	247.68 ± 1.67^gh^
	20:80	7485.0 ± 8.38^c^	575.77 ± 1.48^c^	21.49 ± 0.25^hi^	131.6 ± 0.27^d^	73.01 ± 0.39^ef^	260.25 ± 1.15^ef^
	10:90	8562.5 ± 3.84^b^	658.62 ± 1.37^b^	23.64 ± 0.59^g^	156.89 ± 0.63^b^	77.45 ± 0.46^cd^	289.74 ± 1.63^c^
	0:100	9530.0 ± 3.11^a^	733.08 ± 0.79^a^	25.90 ± 0.18^ef^	186.50 ± 0.11^a^	81.70 ± 0.98^ab^	334.67 ± 1.60^a^
Unpeeled _500 μm sieve particle size	90:10	1183.1 ± 5.66^s^	91.00 ± 0.44^s^	6.35 ± 0.17^r^	51.47 ± 0.17^no^	41.37 ± 0.13^s^	127.11 ± 1.87^qr^
	80:20	2310.7 ± 9.90^q^	177.69 ± 0.76^q^	8.59 ± 0.24^q^	64.13 ± 0.21^kl^	43.91 ± 0.18^rs^	140.47 ± 1.13^p^
	70:30	3165.1 ± 2.21^o^	243.47 ± 1.63^o^	10.63 ± 0.19^pq^	73.90 ± 0.27^ij^	47.63 ± 0.25^opq^	171.99 ± 1.58^mn^
	60:40	3791.5 ± 5.66^l^	291.62 ± 0.43^l^	13.46 ± 0.27^no^	86.21 ± 0.24^h^	52.06 ± 0.16^mn^	183.49 ± 2.96^l^
	50:50	4880.0 ± 6.97^j^	375.39 ± 1.31^j^	15.07 ± 0.38^mn^	94.07 ± 0.25^g^	57.14 ± 0.67^k^	215.43 ± 1.00^j^
	40:60	5569.1 ± 8.30^h^	428.39 ± 0.19^h^	16.93 ± 0.53^lm^	104.38 ± 0.59^f^	61.58 ± 0.39^ij^	233.45 ± 1.76^i^
	30:70	6805.0 ± 9.90^e^	523.46 ± 0.76^e^	18.60 ± 0.45^kl^	120.16 ± 0.27^e^	68.45 ± 0.44^g^	252.86 ± 1.47^fg^
	20:80	7492.0 ± 8.38^c^	576.31 ± 1.44^c^	20.88 ± 0.58^hij^	132.11 ± 0.61^d^	73.89 ± 0.25^e^	264.85 ± 1.95^de^
	10:90	8635.0 ± 5.66^b^	664.23 ± 0.84^b^	22.97 ± 0.79^gh^	157.30 ± 0.42^b^	78.60 ± 0.14^bc^	292.84 ± 1.39^c^
	0:100	9570.0 ± 5.46^a^	736.16 ± 1.96^a^	25.10 ± 0.45^efg^	187.18 ± 0.18^a^	83.21 ± 0.16^a^	335.82 ± 2.65^a^

Values in the same column having no superscript letter in common are significantly different at (*p* < 0.05). TPC = total phenolic content, TFC = total flavonoid content, TAA = total antioxidant activity and RAE = retinol activity equivalent. Values are expressed as mean ± standard deviation (*n* = 3).

**Table 4:** Effect of sieve particle size and flour proportion on the pasting properties of peeled and unpeeled OFSP composite flours.

OFSP Flour Processing	Wheat: OFSP Flour (%)	Peak Viscosity (cP)	Trough Viscosity (cP)	Breakdown Viscosity (cP)	Final Viscosity (cP)	Setback Viscosity (cP)	Peak Time (min)	Pasting Temperature (°C)
Peeled_250 μm sieve particle size	100:0	1501.0 ± 1.2^a^	801.0 ± 1.5^a^	700.0 ± 0.8^a^	1858.0 ± 0.3^a^	1057.0 ± 1.7^a^	5.74 ± 0.01^a^	86.52 ± 0.02^a^
	90:10	640.0 ± 1.1^c^	232.5 ± 0.9^c^	407.5 ± 0.4^c^	620.5 ± 0.8^c^	388.0 ± 0.8^c^	5.01 ± 0.01^c^	82.74 ± 0.01^cd^
	80:20	422.0 ± 1.6^e^	139.5 ± 1.4^e^	282.5 ± 0.0^d^	341.5 ± 0.7^e^	202.0 ± 1.3^e^	4.81 ± 0.01^e^	82.45 ± 0.01^d^
	70:30	289.5 ± 1.4^g^	109.5 ± 1.2^f^	180.0 ± 1.4^f^	219.5 ± 0.4^f^	110.0 ± 0.6^f^	4.75 ± 0.01^f^	82.33 ± 0.01^d^
	60:40	234.0 ± 0.9^j^	79.5 ± 0.7^g^	154.5± 0.0^gh^	139.5 ± 0.0^g^	60.0 ± 0.3^g^	4.36 ± 0.01^h^	82.82 ± 0.02^cd^
	50:50	208.5 ± 1.5^l^	55.5 ± 1.2^hi^	153.0 ± 0.0^h^	93.5 ± 0.5^i^	38.0 ± 0.8^h^	4.17 ± 0.01^jkl^	82.27 ± 0.01^d^
	40:60	197.0 ± 1.7^m^	51.5 ± 1.1^jk^	145.5 ± 0.6^i^	75.5 ± 0.5^j^	24.0 ± 0.4^ij^	4.11 ± 0.02^no^	82.25 ± 0.00^d^
	30:70	154.5 ± 2.2^o^	45.0 ± 1.7^m^	109.5 ± 0.7^k^	67.5 ± 1.0^k^	22.5 ± 0.3^jk^	4.05 ± 0.00^p^	82.24 ± 0.00^d^
	20:80	149.0 ± 0.0^pq^	38.5 ± 0.0^opq^	110.5 ± 0.0^k^	59.5 ± 0.7^l^	21.0 ± 0.0^k^	4.13 ± 0.01^mn^	83.22 ± 0.01^c^
	10:90	139.5 ± 1.8^rs^	35.5 ± 0.9^pqrs^	104.0 ± 0.0^lm^	53.5 ± 0.3^n^	18.0 ± 1.0^l^	4.14 ± 0.01^klmn^	83.98 ± 0.01^b^
	0:100	98.0 ± 0.0^v^	34.0 ± 0.2^rs^	64.0 ± 0.0^op^	50.0 ± 0.5^o^	16.0 ± 0.0^lm^	4.14 ± 0.00^klmn^	83.98 ± 0.01^b^
Peeled_500 μm sieve particle size	100:0	1500.5 ± 0.8^a^	800.5 ± 0.7^a^	700.0 ± 0.4^a^	1858 ± 0.3^a^	1057.5 ± 0.6^a^	5.73 ± 0.01^a^	86.50 ± 0.00^a^
	90:10	639.0 ± 1.3^c^	231.0 ± 0.8^c^	408.0 ± 0.0^c^	621.5 ± 0.3^c^	390.5 ± 1.2^c^	5.02 ± 0.01^c^	82.73 ± 0.01^cd^
	80:20	420.5 ± 1.4^e^	139.0 ± 1.3^e^	281.5 ± 0.4^d^	342.0 ± 0.9^e^	203.0 ± 0.8^e^	4.83 ± 0.00^de^	82.43 ± 0.00^d^
	70:30	289.0 ± 0.0^g^	108.5 ± 0.3^f^	180.5 ± 0.3^f^	221.0 ± 1.1^f^	112.5 ± 0.7^f^	4.76 ± 0.00^f^	82.31 ± 0.01^d^
	60:40	235.0 ± 0.8^ij^	78.5 ± 0.0^g^	156.5 ± 0.7^g^	141.5 ± 0.8^g^	63.0 ± 0.0^g^	4.37 ± 0.01^h^	82.81 ± 0.03^cd^
	50:50	208.0 ± 0.0^l^	54.5 ± 0.7^hij^	153.5 ± 0.5^h^	94.5 ± 0.0^hi^	40.0 ± 0.3^h^	4.17 ± 0.01^ijk^	82.27 ± 0.00^d^
	40:60	196.5 ± 1.0^m^	50.5 ± 0.2^kl^	146.0 ± 0.0^i^	76.5 ± 0.6^j^	26.0 ± 0.7^i^	4.13 ± 0.01^mn^	82.24 ± 0.01^d^
	30:70	153.5 ± 1.6^op^	44.5 ± 1.5^mn^	109.0 ± 0.3^kl^	69.0 ± 1.5^k^	24.5 ± 0.7^ij^	4.06 ± 0.01^p^	82.22 ± 0.00^d^
	20:80	148.0 ± 0.8^q^	37.5 ± 0.0^opqr^	110.5 ± 0.0^k^	60.5 ± 0.7^l^	23.0 ± 0.0^jk^	4.14 ± 0.01^klmn^	83.21 ± 0.01^c^
	10:90	138.5 ± 0.7^rs^	35.0 ± 0.6^qrs^	103.5 ± 0.7^m^	54.5 ± 0.9^n^	19.5 ± 0.3^l^	4.15 ± 0.01^klmn^	83.95 ± 0.01^b^
	0:100	96.5 ± 0.4^v^	33.5 ± 0.0^s^	63.0 ± 0.3^p^	51.0 ± 0.0^o^	17.5 ± 0.5^lm^	4.15 ± 0.01^klm^	83.97 ± 0.01^b^
Unpeeled_250 μm sieve particle size	90:10	742.0 ± 1.5^b^	289.5 ± 1.3^b^	452.5 ± 0.7^b^	808.5 ± 0.6^b^	519.0 ± 0.6^b^	5.13 ± 0.01^b^	82.41 ± 0.01^d^
	80:20	429.0 ± 1.3^d^	143.5 ± 1.0^d^	285.5 ± 1.4^d^	359.5 ± 0.8^d^	216.0 ± 0.5^d^	5.01 ± 0.01^c^	82.36 ± 0.01^d^
	70:30	301.0 ± 1.1^f^	111.5 ± 1.1^f^	189.5 ± 1.1^e^	222.5 ± 1.1^f^	111.0 ± 0.4^f^	4.87 ± 0.01^d^	82.31 ± 0.01^d^
	60:40	239.5 ± 2.0^h^	81.5 ± 1.3^g^	158.0 ± 0.0^g^	142.0 ± 0.4^g^	60.5 ± 0.7^g^	4.41 ± 0.01^g^	82.30 ± 0.01^d^
	50:50	214.0 ± 1.4^kl^	58.0 ± 1.4^h^	156.0 ± 0.0^gh^	95.5 ± 0.7^hi^	37.5 ± 0.3^h^	4.21 ± 0.01^i^	82.25 ± 0.00^d^
	40:60	199.0 ± 1.4^m^	53.5 ± 1.2^ijk^	145.5 ± 0.4^i^	77.5 ± 0.5^j^	24.0 ± 1.0^ij^	4.14 ± 0.01^klmn^	82.25 ± 0.01^d^
	30:70	165.5 ± 0.7^n^	47.0 ± 0.3^lm^	118.5 ± 0.0^j^	68.5 ± 0.6^k^	21.5 ± 0.1^k^	4.08 ± 0.01^op^	82.23 ± 0.00^d^
	20:80	152.0 ± 1.5^pq^	41.0 ± 1.0^no^	111.0 ± 0.0^k^	61.5 ± 0.3^l^	20.5 ± 0.3^k^	4.14 ± 0.01^klmn^	83.21 ± 0.01^c^
	10:90	143.0 ± 1.2^r^	39.0 ± 0.9^op^	104.0 ± 0.3^lm^	57.5 ± 0.2^m^	18.5 ± 0.2^l^	4.14 ± 0.01^klmn^	83.96 ± 0.01^b^
	0:100	108.5 ± 1.1^t^	36.0 ± 1.1^pqrs^	72.5 ± 0.7^n^	51.0 ± 1.1^o^	15.0 ± 0.0^m^	4.14 ± 0.01^klmn^	83.96 ± 0.0^b^
Unpeeled_500 μm sieve particle size	90:10	741.0 ± 1.1^b^	288.5 ± 1.0^b^	452.5 ± 1.2^b^	809.5 ± 0.3^b^	521.0 ± 0.6^b^	5.13 ± 0.0^b^	82.40 ± 0.00^d^
	80:20	428.5 ± 1.4^d^	142.5 ± 1.4^de^	286.0 ± 0.0^d^	361.5 ± 0.7^d^	219.0 ± 0.4^d^	5.02 ± 0.00^c^	82.35 ± 0.01^d^
	70:30	299.0 ± 1.1^f^	110.5 ± 0.8^f^	188.5 ± 0.5^e^	222.5 ± 0.9^f^	112.0 ± 0.0^f^	4.85 ± 0.01^d^	82.29 ± 0.01^d^
	60:40	235.5 ± 0.9^hi^	80.0 ± 0.0^g^	155.5 ± 0.0^gh^	143.0 ± 1.4^g^	63.0 ± 0.3^g^	4.41 ± 0.01^g^	82.28 ± 0.01^d^
	50:50	211.0 ± 0.5^kl^	56.5 ± 0.5^hi^	154.5 ± 0.4^gh^	97.5 ± 0.2^h^	41.0 ± 0.9^h^	4.20 ± 0.01^ij^	82.23 ± 0.01^d^
	40:60	197.5 ± 0.7^m^	53.0 ± 0.4^ijk^	144.5 ± 1.2^i^	78.5 ± 0.4^j^	25.5 ± 0.7^ij^	4.13 ± 0.01^lmn^	82.24 ± 0.01^d^
	30:70	164.5 ± 1.0^n^	45.5 ± 0.2^m^	119.0 ± 1.4^j^	67.5 ± 0.5^k^	22.0 ± 0.0^k^	4.07 ± 0.0^p^	82.23 ± 0.01^d^
	20:80	150.5 ± 0.8^opq^	38.5 ± 0.7^opq^	112.0 ± 0.0^k^	59.5 ± 0.7^l^	21.0 ± 0.2^k^	4.13 ± 0.01^lmn^	83.20 ± 0.00^c^
	10:90	142.0 ± 1.5^r^	37.0 ± 1.3^pqrs^	105.0 ± 0.6^lm^	56.5 ± 0.6^m^	19.5 ± 0.5^kl^	4.17 ± 0.0^ijk^	83.93 ± 0.01^b^
	0:100	106.5 ± 1.2^t^	35.0 ± 1.1^qrs^	71.5 ± 0.8^n^	52.0 ± 1.1^o^	17.0 ± 0.0^lm^	4.12 ± 0.01^mno^	83.90 ± 0.00^b^

Values in the same column having no superscript letter in common are significantly different at (*p* < 0.05). Values are expressed as mean ± standard deviation (*n* = 2).
